# Surgical approach to coexistent inguinal endometriosis and inguinal hernial defect: a case report

**DOI:** 10.1093/jscr/rjad229

**Published:** 2023-08-04

**Authors:** Nouf Almagushi, Leen Almadhi, Nadia Aljomah, Ibrahim Albabtain

**Affiliations:** College of Medicine, King Saud bin Abdulaziz University for Health Sciences, 11362 Riyadh, Saudi Arabia; College of Medicine, King Saud bin Abdulaziz University for Health Sciences, 11362 Riyadh, Saudi Arabia; Trauma and Acute Care Unit, Department of Surgery, King Saud University Medical City, 11362,Riyadh, Saudi Arabia; Department of Surgery, Ministry of the National Guard-Health Affairs, King Saud bin Abdulaziz University for Health Sciences, King Abdullah International Medical Research Center, 11362 Riyadh, Saudi Arabia

**Keywords:** inguinal hernia, endometriosis, cyst

## Abstract

The presence of ectopic endometrial and myometrial tissue is referred to as endometriosis. The majority of cases of inguinal endometriosis are linked to prior uterine surgery. We present a 39-year-old female presented to the general surgery clinic in June 2013 with mild right inguinal pain and swelling. Enhanced computed tomography scan of the abdominal and pelvis revealed an elongated cystic mass in the right groin region. Diagnostic laparoscopy was performed and the uterus, both ovaries, and pouch of Douglas were examined. Two endometrial deposits were detected: on in the superoposteriar part of the uterus and another below the base of the right fallopian tube. In addition to the right inguinal hernia, a cystic lesion was noted on the surface of the round ligament. The entire round ligament, including the canal of Nuck, and cyst were dissected and excised completely. The right inguinal hernia was repaired.

## INTRODUCTION

The presence of ectopic endometrial and myometrial tissue is referred to as endometriosis. In most cases, the stroma and epithelium are visible together; occasionally, only one component is present [1].With an estimated prevalence of 1–15%, > 80% of all women are of reproductive age [2, 3]. The ovary and the peritoneum are frequently affected.

Approximately 1% of all patients diagnosed with endometriosis may develop non-cutaneous inguinal endometriosis secondary to extraperitoneal involvement of the round ligament [4].

Inguinal endometriosis commonly presents as a painful inguinal mass that resembles a hernia; the right side is often involved. In certain situations, the pain and tenderness associated with the mass can worsen during menstruation (catamenial aggravation). Furthermore, most cases of inguinal endometriosis are associated with prior uterine surgery [5].

This presentation is rare; only six patients with inguinal endometriosis have been recorded in four published reports [6].

Here, we present the case of a 39-year-old female who was incidentally found to have right-sided inguinal endometriosis, with no prior history of gynecologic surgery.

## PRESENTATION OF THE CASE

A 39-year-old female who was medically and surgically free of complications presented to the general surgery clinic in June 2013 with mild right-sided inguinal pain and swelling.

Clinical examination did not reveal an inguinal hernia. Diagnostic imaging, including inguinal ultrasonography, revealed an area of hypodensity in the right pubic region, without a hernia or hernial sac.

Enhanced CT scans of the abdomen and pelvis were obtained, which revealed an elongated cystic mass in the right groin. The patient was reassured.

After 9 years, the patient revisited the general surgery clinic complaining of persistent right inguinal pain and enlargement of the swelling.

Physical examination revealed a soft abdomen with a cystic lesion medial to the pubic bone; cough impulse was negative. No signs of peritonitis or inguinal hernia were noted.

Laboratory workup, such as complete blood count (CBC) and cancer antigen 125 (CA125) level estimation, were performed and were unremarkable.

**Figure 1:**
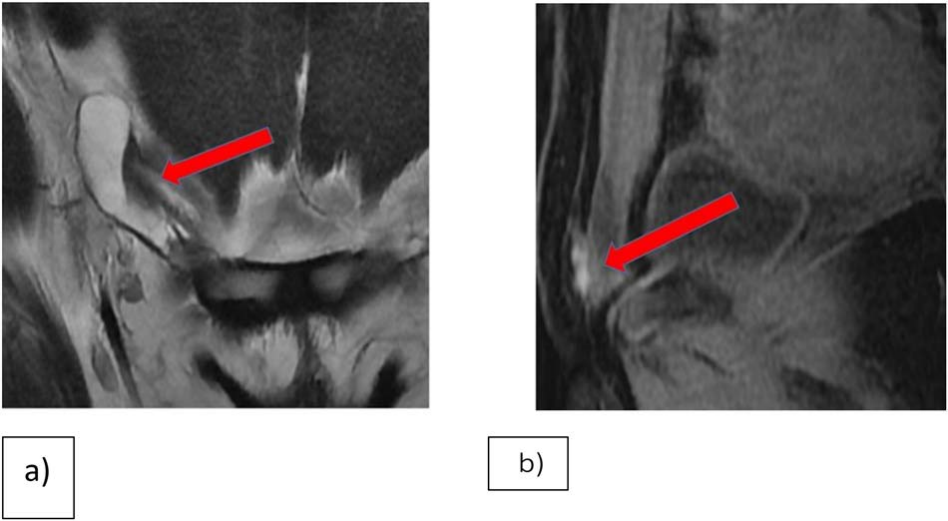
(**a**) Coronal T2WI showing right inguinal tubular cystic lesion that is communicating with peritoneum highly suggestive of communicating hydrocele of canal of Nuck. (**b**) MRI Sagittal T1 fat saturation, demonstrating that the medial superior aspect of the cyst is bright on T1WI in keeping with hemorrhagic contents versus endometrial implant.

Enhanced CT-scans of the abdomen and pelvis demonstrated an increase in the size of the elongated cystic lesion. A multiplanar multi-sequential MRI of the pelvis revealed a right inguinal lesion, likely an endometrial plaque with hydrocele of the canal of Nuck (Fig. 1).

A gynecologist was consulted to confirm the diagnosis of endometriosis, which was excluded based on clinical and radiological evaluations.

Diagnostic laparoscopy was performed, with three small incisions with one 12- and two 5-mm ports supra-umbilical and a right and left midclavicular line ports).

**Figure 2:**
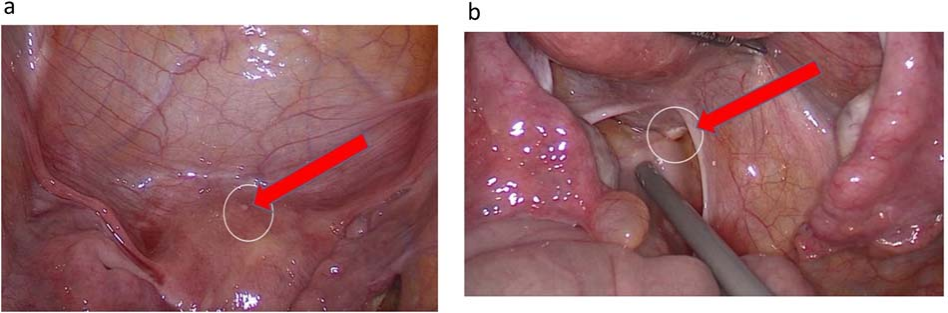
(**a**) Endometrial lesion in superoposteriar uterus. (**b**) Endometrial lesion in the base of right Fallopian tube.

**Figure 3:**
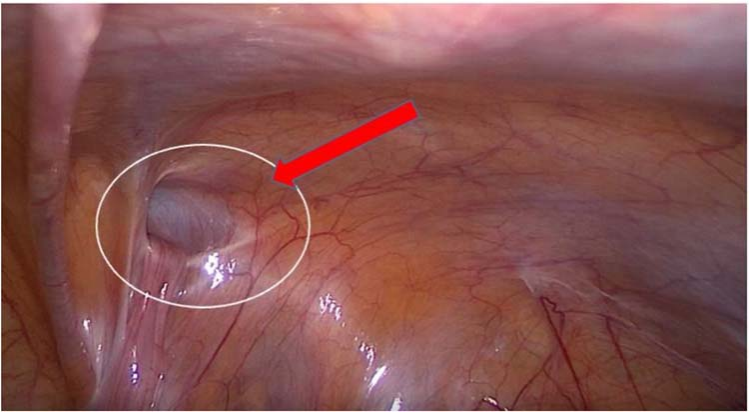
Transabdominal prepertoneal (TAPP) view of right inguinal defect.

**Figure 4:**
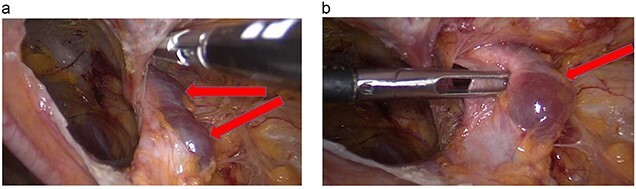
(**a**, **b**) Cyst over the right round ligament.

The uterus, both ovaries, and pouch of Douglas were examined. Two endometrial deposits were detected: one the anteroposterior part of the uterus and another below the base of the right fallopian tube. In addition, there was right inguinal hernial defect and a cystic lesion on the surface of the round ligament (Figs 2–4).

**Figure 5:**
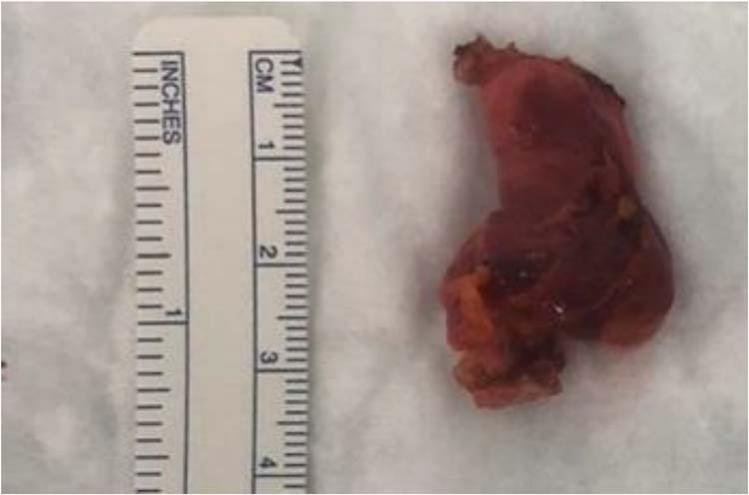
Right round ligament with the cystic lesion.

A transabdominal preperitoneal (TAPP) approach was used. The entire round ligament, including the canal of Nuck, and cyst were dissected and excised completely (Fig. 5).

The inguinal hernia was repaired using a biosynthetic mesh (Phasix ST; Becton and Dickinson company, Franklin Lakes, United States).

Pathological examination revealed areas of focal endometrial parenchyma in the specimen, with no evident atypical features or neoplasia.

The patient had an unremarkable postoperative course. She was followed up in the clinic. She did not have any complaints and was on oral contraceptives for hormonal suppression.

The final pathology was reported as a focal endometriosis with no evidence of atypical features or neoplasia.

## DISCUSSION

Endometriosis is characterised by the presence of endometrial glands and stroma at extrauterine locations. Endometriosis can affect practically any organ system; however, the majority of ectopic implants are found in the pelvis.

Extra-pelvic endometriosis can occur at multiple sites, including the gastrointestinal and urinary tracts, thoracic cavity, central nervous system, umbilicus, skin (abdominal wall incisions and episiotomy scars), and inguinal canal.Extra-pelvic endometriosis is associated with a variety of unusual symptoms, including pain and physiological dysfunction [7].

Inguinal endometriosis, which may affect 0.3–0.6% of patients with endometriosis, is identified by the occurrence of endometrial stroma and glands in the extraperitoneal regions of the round ligament and surrounding connective and lymphatic tissues [7].

Patients usually present with a tender inguinal swelling during their reproductive years, with a higher prevalence between 30 and 40 years of age [8]. Due to the difficulty in making a diagnosis, inguinal endometriosis is frequently mistaken for more prevalent illnesses, such as hernias, lymphadenopathy, granulomas, soft tissue tumors, cysts and hydroceles. Approximately 50% of patients have catamenial symptoms, which should raise the index of suspicion for inguinal endometriosis; the mass can vary in size and tenderness [9].

Inguinal endometriosis occurs on the right side in >90% of cases and is infrequently observed with occult hernias [10]. Occasionally, inguinal endometriosis can coexist with inguinal hernias and other groin diseases, making the diagnosis more challenging. A groin hernia and inguinal endometriosis coexisted in 37% of participants in one study [9].

Endometriosis must be distinguished from other inguinal masses prior to surgery using CT, magnetic resonance imaging or ultrasonography.

Ultrasonography is the primary modality for diagnosing inguinal endometriosis and locating concurrent hernia sacs. Inguinal endometriosis can manifest on ultrasounds in several ways, including solid masses, cystic masses, and mixed cystic and solid masses [11]. MRI must be used to detect subperitoneal endometriotic deposits and diagnose extraperitoneal abnormalities.

Surgical excision is the preferred method of treatment for inguinal endometriosis. Removal of the extraperitoneal part of the round ligament is locally curative and must be performed. The complete removal of the lesion enables histological diagnosis and prevents recurrence. To further assess lesions and the extent of endometriosis, diagnostic laparoscopy or postoperative referral to a gynecologist is necessary, given the high concordance of pelvic endometriosis.

Furthermore, oral contraceptives or GnRH agonists may occasionally be used to suppress hormone levels.

## CONCLUSION

This report aimed to encourage medical professionals to consider inguinal endometriosis as a differential diagnosis in reproductive-age women who are diagnosed with congenital cystic lesions (canal of Nuck cyst) and perform diagnostic workups to rule it out.

## CONSENT

Written informed consent was obtained from the patient for publication of this case report and accompanying images. A copy of the written consent is available for review by the Editor-in-Chief of this journal on request.

## CONFLICT OF INTEREST STATEMENT

None declared.

## FUNDING

None.
